# Comparison analysis of serum interleukin-6 levels and cervical cancer

**DOI:** 10.3332/ecancer.2025.2009

**Published:** 2025-10-07

**Authors:** Muisi A Adenekan, Joseph B Minari, Ayodeji Adefemi, Gbenga Olorunfemi, Ayomide I Fayinto, Adebayo Sekumade, Temitope V Adekanye, Adeyemi A Okunowo, Kehinde S Okunade

**Affiliations:** 1 Department of Obstetrics and Gynaecology, Lagos Island Maternity Hospital, Lagos 102273, Nigeria; 2 Genome Editing Research Group, Department of Cell Biology and Genetics, University of Lagos, Lagos 101017, Nigeria; 3 Department of Obstetrics and Gynaecology, Lagos State University Teaching Hospital, Lagos 101233, Nigeria; 4 Division of Epidemiology and Biostatistics, School of Public Health, University of Witwatersrand, Johannesburg 2193, South Africa; 5 Department of Obstetrics and Gynaecology, Nnamdi Azikiwe University Teaching Hospital, Nnewi 435101, Nigeria; 6 Department of Obstetrics and Gynaecology, Lagos University Teaching Hospital, Lagos 102215, Nigeria; 7 Department of Obstetrics and Gynaecology, College of Medicine, University of Lagos, Lagos 102215, Nigeria; 8 Center for Clinical Trial Research and Implementation Science, College of Medicine, University of Lagos, Lagos 102215, Nigeria

**Keywords:** cervical cancer, inflammation, interleukin-6, Nigeria, prognosis

## Abstract

**Synopsis:**

We found a significant increase in serum IL-6 levels of 214.9 ng/mL (95% CI: 60.1–369.7, *p* = 0.007) among women with cervical cancer (CC) compared to their cancer-free counterparts.

**Objectives:**

Despite growing evidence of the role of interleukin-6 (*IL-6*) in CC, studies assessing this association among women of sub-Saharan African origin remain limited. This study investigated the association between serum *IL-6* and CC and explored the effects of serum IL-6 levels on prognosis in patients with CC in Lagos, Nigeria.

**Methods:**

We conducted a cross-sectional study among women with and without CC in two hospitals. A venous blood sample was collected from each participant for laboratory analysis of serum *IL-6* levels. We performed simple and multiple linear regression analyses to compare the unit increase in serum *IL-6* levels between study groups while adjusting for relevant confounders.

**Results:**

Our study found a significant unit increase of 214.9 ng/mL (95%CI: 60.1–369.7, *p* = 0.007) in serum *IL-6* levels among women with CC compared to their cancer-free counterparts. The area under the curve of 0.814 demonstrated a good discriminatory ability at an optimal serum IL-6 cut-off value of 365.1 ng/mL. *IL-6* levels were significantly elevated in patients with advanced-stage CC compared to those with early-stage disease (156.7 (IQR: 130.4–227.6) versus 324.7 (IQR: 188.5–516.2) ng/mL) and in patients diagnosed with squamous cells carcinoma (SCC) compared to those without SCC (523.9 (IQR: 365.1–682.1) versus 203.6 (IQR: 131.3–334.3) ng/mL).

**Conclusion:**

Our study findings highlight the potential utility of serum *IL-6* as a biomarker for CC diagnosis and prognosis. However, further studies are needed to explore the utility of *IL-6* as a target for therapeutic intervention and in the treatment monitoring of CC patients.

## Introduction

Cervical cancer (CC) remains a significant public health challenge worldwide [1], with its burden disproportionately affecting low- and middle-income countries [2]. Data indicate that sub-Saharan Africa bears the highest incidence and mortality rates from the disease [1, 3, 4], with Nigeria alone reporting approximately 12,075 new cases and 10,403 deaths annually [2, 5].

The development of CC has been strongly linked to persistent infection with oncogenic human papillomavirus (HPV), the primary etiological agent of the disease and its precursor lesions [6, 7]. However, the progression from HPV infection to invasive cancer is influenced by a complex interplay of environmental, lifestyle and host factors that modulate viral activity [1, 8]. Individual genetic variations in immune mediators play a critical role in susceptibility to neoplasia [9], with increasing evidence suggesting that pro-inflammatory cytokines contribute to CC pathogenesis [10].

Interleukin-6 (*IL-6*) is a multifunctional cytokine secreted by various cell types, such as macrophages, T cells, fibroblasts and endothelial cells, in response to infections, tissue damage and a range of inflammatory triggers [11, 12]. It is involved in immune regulation, inflammation, haematopoiesis and cancer development and progression [12]. *IL-6* has been implicated in tumourigenesis by promoting cell proliferation, angiogenesis and resistance to apoptosis in several cancers [13–15], including CC, and is now considered a critical biomarker and therapeutic target in various diseases, particularly in inflammation-related pathologies and cancer [11, 13–15]. However, despite growing evidence of its role in CC [16–18], studies assessing the association between *IL-6* and CC among women of sub-Saharan African origin remain limited. To address this gap and provide valuable insights into the role of *IL-6* in CC susceptibility and progression, our study investigated the association between serum *IL-6* and CC and then explored the effects of serum IL-6 levels on prognosis in patients with CC in Lagos, Nigeria.

## Materials and methods

### Study design and setting

This is a cross-sectional study conducted between January and December 2023 at the gynaecological oncology clinics of Lagos State University Teaching Hospital (LASUTH), Ikeja and Lagos Island Maternity Hospital (LIMH), Lagos Island, Lagos, Nigeria. The hospitals serve as public referral centres for other medical facilities in Lagos and its adjoining states. They both run gynaecology, cytology and colposcopy clinics from Monday to Friday of each week, providing evaluation, diagnosis and appropriate treatment for patients with pre-invasive and invasive CC.

### Study population

Eligible participants were women aged 18 years or more with histologically confirmed CC (case group) enrolled from the study clinics using the consecutive sampling technique. The comparison group comprised randomly selected, unmatched healthy and cancer-free women attending the gynaecological and cytology clinics during the same study period as the case group. Exclusion criteria included a history of other malignancies, withdrawal of consent during the study and lack of physical and mental capacity precluding understanding of informed consent.

### Study procedures

On confirming each participant’s eligibility, informed written consent was obtained upon explanation of the goals and procedures of the study. Information on sociodemographic and clinical characteristics, including participants’ age, parity, marital (married or unmarried) and menopausal (pre- and post-menopausal) status, types of marriage (mono- or polygamous), educational, occupational and socioeconomic status, religion, ethnicity, initiation of sexual activity and age at coitarche, use of alcoholic beverages and oral contraceptive pills, number of lifetime sex partners and International Federation of Gynaecology and Obstetrics (FIGO) stage and histological type of disease, were obtained from each participant, after which about 5 mL of peripheral blood samples were collected through venipuncture into a plain specimen bottle (without anticoagulant), sealed and transported within an hour to the main laboratory at LIMH. The samples were centrifuged to separate the serum, which was then stored frozen at −80°C before batched analysis of serum *IL-6* concentrations with Human interleukin-6 ELISA Kit (E0090Hu) (Thermo Fisher Scientific Inc., Waltham, MA, USA).

### Sample size calculation

Using the sample size formula for comparing means in Stata, we determined that a total sample size of *n* = 58 would provide 80% power at a type 1 error rate of 5% with provision for a 20% non-response or data recording error rate. This calculation was based on mean *IL-6* values of 23.16 ± 19.57 pg/mL for CC patients and 9.91 ± 9.25 pg/mL for non-CC participants, derived using Hozo’s method [19] from the median *IL-6* values reported in the study by Lippitz and Harris [20].

### Statistical analysis

Statistical analyses were performed using Stata version 18.0 for Windows (StataCorp LLC, TX, USA). Data were summarised using descriptive statistics with continuous variables presented as mean (standard deviation) for normally distributed data or median (25th—75th centile) for skewed distributions. Serum *IL-6* levels between the women with and without CC were compared using the non-parametric Wilcoxon rank sum test. We performed simple (bivariable) and multiple (multivariable) linear regression analyses with a backward conditional approach to compare the unit increase in serum *IL-6* levels between the study groups while adjusting for potential confounders such as participants’ age, menopausal status, age at coitarche and the number of lifetime sex partners using coefficient (*b*) at 95% confidence interval (95%CI). We then performed a receiver operating characteristic (ROC) curve analysis for serum *IL-6* to derive an optimal discriminatory cut-off value for serum *IL-6* levels that distinguished between CC and non-cancer. Subgroup analyses were also performed to compare the serum *IL-6* levels between cancer stage (early versus advanced) and histological type squamous cell carcinoma (SCC versus non-SCC) in women with CC. Statistical significance was reported at *p* < 0.05.

### Statement of ethics

This study was approved by the Health Research Ethics Committee of the LASUTH, the Health Service Commission and the LIMH (approval number LREC/10/1990). We conducted the study ethically per the World Medical Association Declaration of Helsinki. All the study participants read and signed a written informed consent before enrolment, and we ensured strict adherence to the privacy and confidentiality of participants' information during and after conducting the study.

## Results

A total of 107 potential participants were screened for eligibility in the study (58 in LASUTH and 49 in LIMH). Of these, 37 (63.8%) were enrolled from LASUTH, while 23 (46.9%) were from LIMH. We included 60 women in the study (30 women diagnosed with CC and 30 cancer-free comparators). The mean age of participants with CC was significantly higher than that of the comparators (52.0 ± 10.0 years versus 42.6 ± 6.7 years, *p* < 0.001). Similarly, the median age at first sexual intercourse (coitarche) was higher among those with CC compared to the cancer-free comparison group (19.5 years (IQR: 18.0–21.0) versus 17.5 years (IQR: 17.0–18.0), *p* = 0.005). Similarly, women with CC also reported a higher median number of lifetime sexual partners (5 (IQR: 3–6) versus 3 (IQR: 2–5), *p* = 0.002). As summarised in Table 1, other sociodemographic characteristics were similar between the groups.

The clinicopathological characteristics of the CC patients (*n* = 30) are summarised in Table 2. Cervical SCC was the most common histological type of CC (70.0%, *n* = 21), while the least common was adeno-squamous carcinoma (6.7%, *n* = 2). Most CC patients presented with advanced disease (36.7%, *n* = 11).

**Figure 1.**
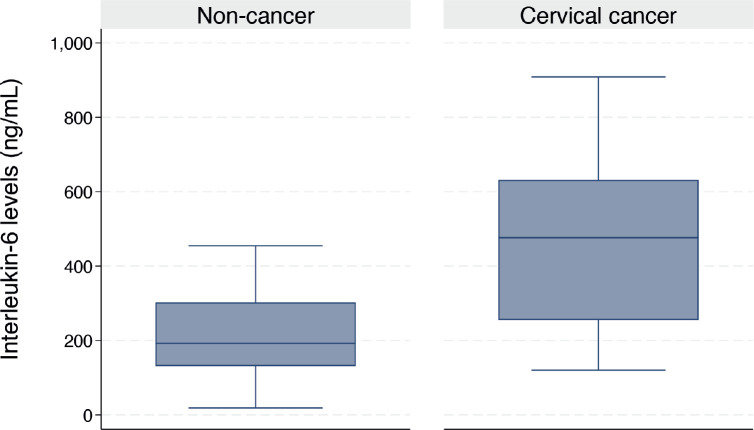
Box plot showing the serum *IL-6* levels in women with and without CC (476.3 (IQR: 254.9–631.9) versus 192.4 (IQR: 131.0–302.4) ng/mL, *p* < 0.001).

**Figure 2.**
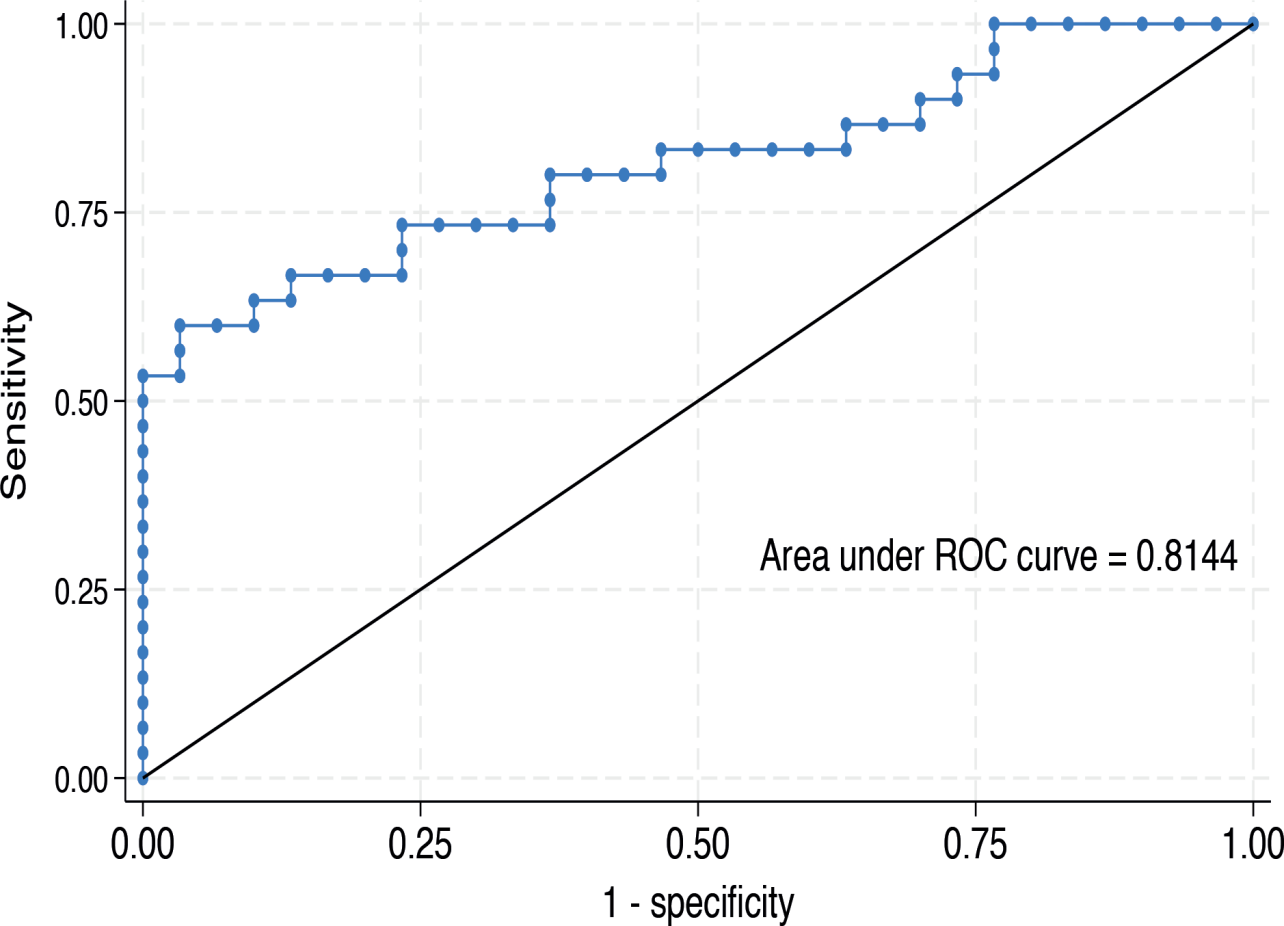
ROC showing an AUC of 0.814 (95%CI 0.704–0.925) at a discriminatory serum *IL-6* cut-off value of 365.1 ng/mL.

The median serum *IL-6* level was higher in women with CC than in their cancer-free counterparts (476.3 (IQR: 254.9–631.9) versus 192.4 (IQR: 131.0–302.4) ng/mL, *p* < 0.001) as shown in Figure 1. In the bivariable linear regression analysis performed in Table 2, CC was significantly associated with increased serum *IL-6* levels (*p* < 0.001). As shown in Table 3, after adjusting for potential confounders in the multivariable model, this association remained statistically significant with an estimated unit change of +214.9 ng/mL (95% CI: 60.1–369.7, *p* = 0.007). Figure 2 shows the ROC curve for serum *IL-6*, demonstrating its discriminatory ability. The area under the curve (AUC) was 0.814 (95%CI: 0.704–0.925), indicating good diagnostic performance. The optimal serum *IL-6* cut-off value for distinguishing between CC patients and their cancer-free counterparts was 365.1 ng/mL, corresponding to a sensitivity of 66.7% and a specificity of 86.7%.

**Figure 3.**
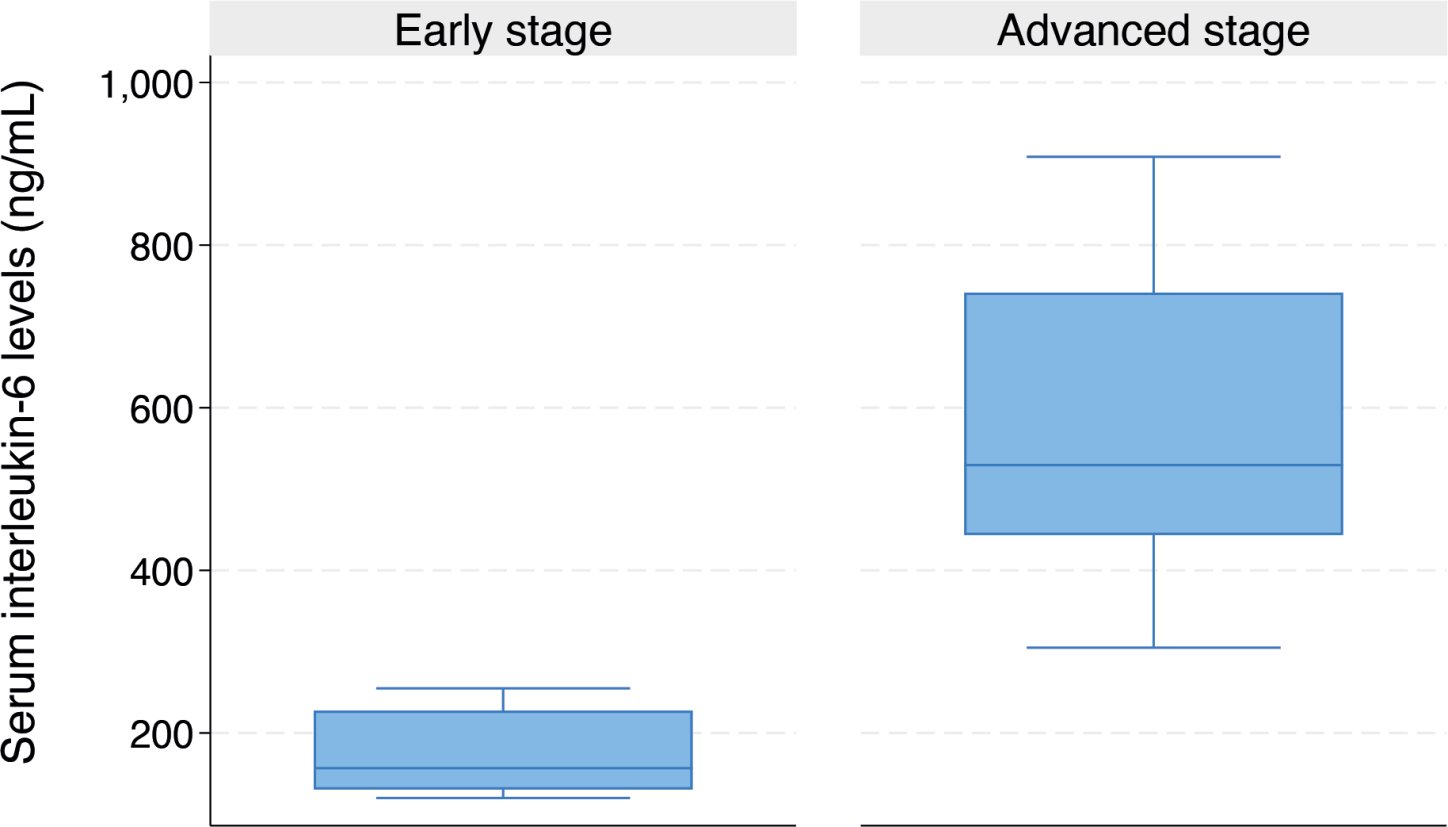
Box plot showing the serum *IL-6* levels in women with early and advanced stage CC (156.7 (IQR: 130.4–227.6) versus 324.7 (IQR: 188.5–516.2) ng/mL, *p* = 0.015).

**Figure 4.**
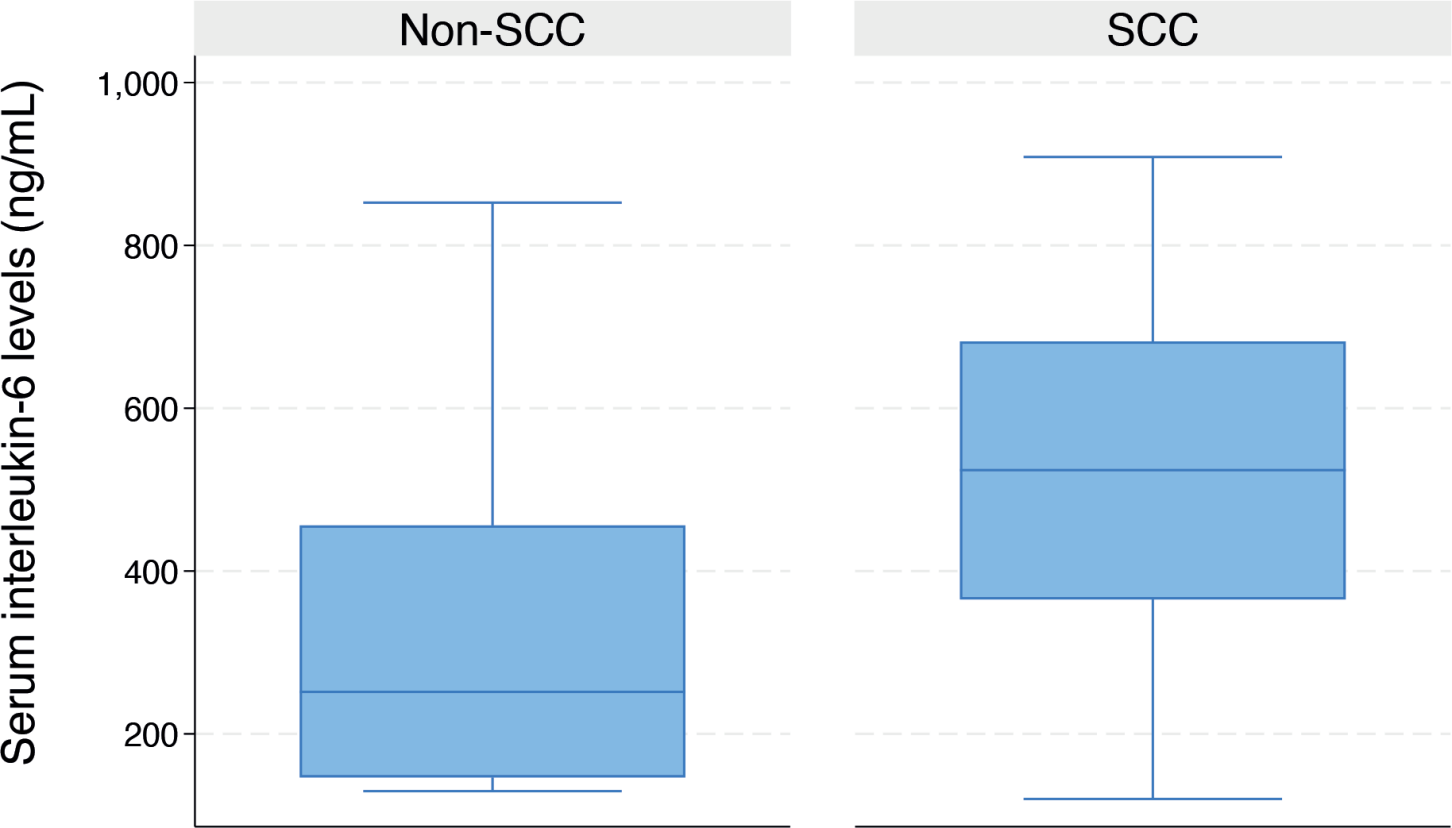
Box plot showing the serum *IL-6* levels in women with or without cervical SCC (523.9 (IQR: 365.1–682.1) versus 203.6 (IQR: 131.3–334.3)).

As illustrated in the box plot in Figure 3, the median serum *IL-6* level was significantly higher in women with advanced-stage CC compared to those with early-stage disease (324.7 ng/mL (IQR: 188.5–516.2) versus 156.7 ng/mL (IQR: 130.4–227.6), *p* = 0.015). The median serum *IL-6* level was significantly higher in women diagnosed with SCC compared to those without SCC (523.9 ng/mL (IQR: 365.1–682.1) versus 203.6 ng/mL (IQR: 131.3–334.3), *p* < 0.001) (Figure 4).

## Discussion

Our study evaluated the association between serum *IL-6* and CC and explored the effects of serum *IL-6* levels on prognosis in patients with CC in Lagos, Nigeria. We reported that serum *IL-6* levels were significantly higher in patients with CC compared to cancer-free women, with an optimal serum *IL-6* cut-off value of 365.1 ng/mL for distinguishing between CC and cancer-free participants. Further stratification of CC patients revealed that *IL-6* levels were significantly elevated in patients with advanced-stage CC compared to those with early-stage disease and in patients diagnosed with SCC compared to those without SCC.

Our study has further provided compelling evidence supporting the association between elevated serum *IL-6* levels and CC in corroboration with the findings by Cai *et al* [16] in Jiangsu, China and Wagh *et al* [21] in Maharashtra, India. This was even similar to the study by Song *et al* [18] in Shanghai, China, which reported a significantly higher *IL-6* expression in formalin-fixed, paraffin-embedded CC tissues than in adjacent non-tumour tissues examined by immunohistochemistry. This further suggests that *IL-6* plays a critical role as a pro-inflammatory cytokine in the tumour microenvironment [22] and its elevated levels in CC patients may reflect ongoing chronic inflammation, which promotes tumour growth, angiogenesis and immune evasion [14]. Persistent infection with high-risk HPV strains, a known driver of cervical carcinogenesis [1], induces inflammatory responses that may contribute to increased *IL-6* expression [23]. Furthermore, similarly to previous studies [16, 21], our study demonstrated a high discriminatory ability for serum *IL-6*, indicating its good diagnostic performance and potential usefulness as a diagnostic adjunct in resource-constrained settings like ours, where access to more advanced diagnostic tools is limited.

As revealed in our study, *IL-6* levels were significantly elevated in patients with advanced-stage CC compared to those with early-stage disease. This finding is consistent with previous studies indicating that *IL-6* plays a critical role in tumour progression, angiogenesis and immune modulation in CC [15, 16, 18, 20, 21]. The immunosuppressive role of *IL-6* contributes to immune evasion by suppressing anti-tumour immune responses, particularly by promoting regulatory T-cell expansion and inhibiting cytotoxic T-cell activity [24]. Additionally, we reported that *IL-6* levels were markedly higher in patients diagnosed with SCC than those without SCC, underscoring its role in the pathophysiology of this predominant and inflammation-driven [25] form of CC. These findings reinforce the role of *IL-6* as a potential biomarker for CC and highlight its likely relevance in the pathogenesis and progression of the disease [24, 26]. Therefore, given its involvement in inflammation and cancer progression [18, 23], *IL-6* could serve as a potential target for therapeutic interventions in CC [11, 14].

The main strength of this study is that it is the first to evaluate the association between serum *IL-6* and the risk of CC in Lagos State, Nigeria. However, the study has a few limitations that should be acknowledged. First, we used a cross-sectional study design, which precludes a definitive conclusion of a causal relationship between IL-6 and CC. Second, the sample sizes used in the subgroup analyses may be insufficient to detect significant associations between *IL-6* and CC stages and histological types. Therefore, future longitudinal studies with large cohorts and diverse populations are necessary to confirm the findings of our study.

## Conclusion

We observed that serum *IL-6* levels were comparably higher in patients with CC than in cancer-free women, giving an optimal discriminatory cut-off value of 365.1 ng/mL. Serum *IL-6* levels were also significantly higher in patients with advanced-stage CC compared to those with early-stage disease and in those diagnosed with SCC compared to those without SCC. These findings highlight the potential utility of serum *IL-6* as a biomarker for CC diagnosis and prognosis. However, further studies are needed to explore *IL-6* as a target for therapeutic intervention and assess its use in risk stratification and treatment monitoring in CC patients.

## Conflicts of interest

The authors have no conflicts of interest.

## Funding

The authors have received no specific funding for this research. However, the author (*KSO*) protected time was supported through funding from the National Cancer Institute and Fogarty International Center of the National Institutes of Health under Award Numbers K43TW011930 and D43TW010934 and the Conquer Cancer International Innovation Grant under Project ID 2024IIG-2761200216. The content of this paper is solely the responsibility of the authors. It does not necessarily represent the official views of the National Cancer Institute, Fogarty International Center or the National Institutes of Health and the Conquer Cancer Foundation. The funding agencies were not involved in the study design, manuscript preparation or the decision to submit the manuscript for publication.

## Author contributions

**Conceptualisation and design:** Muisi A Adenekan, Joseph B Minari

**Provision of study materials or patients:** Muisi A Adenekan, Ayodeji Adefemi, Adebayo Sekumade, Adeyemi A Okunowo, Kehinde S Okunade

**Data collection:** Muisi A Adenekan, Ayodeji Adefemi, Ayomide I Fayinto, Adebayo Sekumade, Temitope V Adekanye

**Data analysis:** Gbenga Olorunfemi, Ayomide I Fayinto, Kehinde S Okunade

**Funding acquisition:** Muisi A Adenekan

**Writing—original draft:** Muisi A Adenekan, Joseph B Minari, Kehinde S Okunade

**Writing—review and editing:** Muisi A Adenekan, Joseph B Minari, Adeyemi A Okunowo, Kehinde S Okunade

**Supervision:** Joseph B Minari, Adeyemi A Okunowo, Kehinde S Okunade

All the authors approved the final version of the manuscript

All authors are accountable for all aspects of the work.

## Data availability

The dataset for this study will be made available on reasonable request from the corresponding author (KSO).

## Figures and Tables

**Table 1.** Comparison of sociodemographic characteristics of participants with and without CC (*n* = 60).

Characteristics	CC	Control	*p*-value
	*n* = 30	*n* = 30	
**Study sites**			
LASUTH	11 (36.7)	29 (96.7)	<0.001
LIMH	19 (63.3)	1 (3.3)	
Menopausal status			
Premenopause	11 (36.7)	29 (96.7)	<0.001
Postmenopause	19 (63.3)	1 (3.3)	
Marital status			
Married	18 (60.0)	16 (53.3)	0.602
Unmarried	12 (40.0)	14 (46.7)	
Types of marriage (*n* = 40)			
Monogamous	12 (52.2)	8 (47.1)	0.749
Polygamous	11 (47.8)	9 (52.9)	
Educational status			
At least primary education	12 (40.0)	11 (36.7)	0.702
Secondary education	14 (46.7)	12 (40.0)	
Tertiary education	4 (13.3)	7 (23.3)	
Occupation			
Housewife	19 (63.3)	16 (53.3)	0.651
Trading	9 (30.0)	6 (20. 0)	
Civil servant	5 (16.7)	5 (16.7)	
Socioeconomic status			
Low	19 (63.3)	19 (63.3)	0.015^
Middle	9 (30.30)	7 (23.3)	
High	2 (6.7)	4 (13.3)	
Religion			
Christianity	19 (63.3)	18 (60.0)	0.791
Muslim	4 (36.7)	12 (40.0)	
Ethnic group			
Yoruba	24 (80.0)	27 (90.0)	0.555^
Igbo	4 (13.3)	2 (6.7)	
Hausa	2 (6.7)	1 (3.3)	
Sexually active			
Yes	30 (100.0)	26 (86.7)	0.038
No	0 (0.0)	4 (13.3)	
Alcoholic drinker			
Yes	3 (10.0)	5 (16.7)	0.448
No	27 (90.0)	25 (83.3)	
OCP usage			
Yes	7 (23.3)	4 (13.3)	0.317
No	23 (76.7)	26 (86.7)	
Mean age ± SD, year	52.0 ± 10.0	42.6 ± 6.7	<0.001
Median parity (IQR)	4 (3–5)	3 (2–4)	0.077^Ω^
Median age of coitarche (IQR), year	19.5 (18.0–21.0)	17.5 (17.0–18.0)	0.005^Ω^
Median lifetime sex partners	5 (3–6)	3 (2–5)	0.002^Ω^

**Abbreviations:** IQR, interquartile range; LASUTH, Lagos State University Teaching Hospital; LIMH, Lagos Island Maternity Hospital; OCP, oral contraceptive pill; SD standard deviation

^ Fischer’s exact test; ^Ω^ Wilcoxon rank sum test

**Table 2.** Clinicopathological characteristics of CC patients (*n* = 30).

Characteristics	Frequency (%)
Histological type	
SCC	21 (70.0)
Adenocarcinoma	7 (23.3)
Adeno-squamous carcinoma	2 (6.7)
FIGO staging	
Stage 1	1 (3.3)
Stage 2	7 (23.3)
Stage 3	11 (36.7)
Stage 4	11 (36.7)

**Abbreviation:** FIGO, International Federation of Obstetrics and Gynaecology

**Table 3.** Linear regression analyses of the association between serum *IL-6* levels and CC.

Variables	Bivariable *p*-value	Multivariable		
		Serum IL-6 levels		*p*-value
		Unit change, *b* (ng/mL)	95% CI	
CC	<0.001	+214.9	60.1–369.7	0.007
Participant’s age, year	0.001	−1.8	–10.3–6.6	0.664
Having at least four children	0.360	NA	NA	NA
Postmenopause	<0.001	80.6	–113.8–275.1	0.409
Married	0.601	NA	NA	NA
Polygamous marriage	0.316	NA	NA	NA
High SES	0.600	NA	NA	NA
Age of coitarche	0.024	7.6	–17.7–32.9	0.548
Alcoholic drinker	0.745	NA	NA	NA
OCP user	0.855	NA	NA	NA
At least four lifetime sex partners	0.042	31.2	–422.8–671.4	0.650

**Abbreviations:** *b* , coefficient; 95%CI, 95% confidence interval; *IL-6,* interleukin-6; NA, not applicable; SES, socioeconomic status; OCP, oral contraceptive pill
